# Thermal degradation assessment of canola and olive oil using ultra-fast gas chromatography coupled with chemometrics

**DOI:** 10.1007/s00706-017-1968-y

**Published:** 2017-07-01

**Authors:** Tomasz Majchrzak, Martyna Lubinska, Anna Różańska, Tomasz Dymerski, Jacek Gębicki, Jacek Namieśnik

**Affiliations:** 1 Department of Analytical Chemistry, Faculty of Chemistry, Gdańsk University of Technology, Gdańsk, Poland; 2 Department of Chemical and Process Engineering, Faculty of Chemistry, Gdańsk University of Technology, Gdańsk, Poland

**Keywords:** Lipids, Food control, Gas chromatography, Oxidations, Vegetable oil

## Abstract

**Abstract:**

Oil blending is often used to enhance the properties of vegetable oils. The admixture of a more thermally stable oil makes the resulting blend more suitable for use in frying. A new method of quality assessment of vegetable oils used in frying is presented in this paper. In this method, ultra-fast gas chromatography coupled with flame ionization detector and chemometrics is employed. Principal component analysis was used for data processing. The results obtained with this method were compared with the results of the Rancimat test and sensory evaluation. It is demonstrated that the addition of olive oil improves the stability of rapeseed oil, and also changes its flavour and aroma profile. In addition, it was found that ultra-fast GC coupled with chemometrics is an effective tool for the assessment of the quality of edible oils. The proposed method does not require sample preparation, and the total time of analysis is less than 2 min.

**Graphical abstract:**

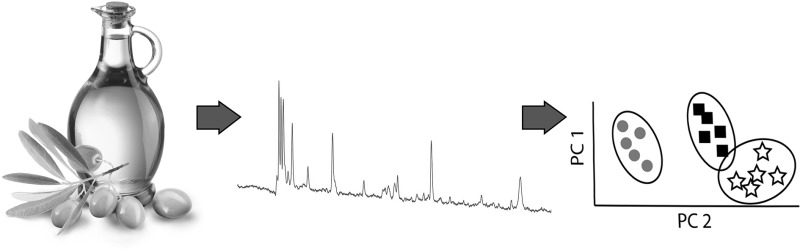

## Introduction

Edible oil may be subjected to various modifications in order to obtain desirable modifications, such as viscosity, flavour, colour, or thermal stability. Such modifications may include hydration or refining. However, the easiest and relatively inexpensive method of improving the parameters of edible oils is blending with other oils [1]. Blending can change the product’s fatty acid composition thereby reducing its reactivity, e.g. during oxidation [2]. In recent years, canola and olive oil blends became commercially available. The admixture of olive oil may improve the flavour and thermal stability of canola oil.

Properly conducted blending of oils of different botanical origins may improve the functional parameters of the final product. For instance, the addition of palm olein, olive oil, and corn oil to canola oil improves its thermal stability [3]. Blending different varieties of vegetable oils may also improve their flavour. It is common practice to add virgin olive oil to odourless refined olive in order to improve its flavour [4]. Mixing edible oils also affects their physical parameters. Addition of liquid sunflower oil to solid palm oil prevents the crystallization of the latter, thereby improving its usability. The change of the physical state is the result of the appearance of unsaturated fatty acids. The presence of these acids also improves the wholesomeness of oil [5].

Frying takes place when the temperature of the oil in which the food is immersed is 150–190 °C. During frying, a number of chemical processes take place. The thermal degradation products can be either volatile or non-volatile chemical compounds. Efficiency of chemical reactions occurring during frying, such as oxidation, hydrolysis, cyclization, polymerization or Millard reaction depends on the oil and fried food composition, as well as on temperature and duration of frying. During frying oxygen can react with the edible oil [2, 6]. The mechanism of oxidation process at high temperatures is similar to autoxidation which occurs during the storage of vegetable oil [2]. The oxidation reaction which occurs during frying is faster than the reaction which proceeds at lower temperatures. The mechanism of oxidation can be divided into three stages: initiation, propagation, and termination. During frying, volatile organic compounds are produced, namely carboxylic acids, hydrocarbons, ketones, aldehydes, esters, lactones, and aromatic compounds [7]. The main VOCs generated during thermal degradation of edible oils are aldehydes such as acrolein and aldehydes olefin, which are dangerous to human health [8]. Products of thermal degradation of vegetable oils can be carcinogenic, may cause diabetes, atherosclerosis, Alzheimer’s and Parkinson’s diseases [9], coronary heart disease [10], sudden cardiac death [11], and systemic vasculitis [12].

The reference methods most commonly used to determine the quality of oil are: sensory analysis [13], measurement of the Totox parameter [14], determination of oxidative stability using the Rancimat test [15], and determination of free fatty acids using gas chromatography [16]. These methods are time-consuming and labour-intensive, and often require sample preparation.

Gas chromatography is a well-established technique dedicated for food control. Due to the need to lessen the time of a single analysis, ultra-fast gas chromatography (ultra-fast GC) is increasingly being used. One advantage of this technique is the possibility of simultaneous separation and identification of chemical compounds in a relatively short time. Presented in this paper is the method for assessing the quality of canola and olive oil blend using ultra-fast GC and chemometrics. The use of fingerprint method allows to obtain reliable results easily and within a short period of time.

## Results and discussion

Sensory panel is commonly used in a direct method of assessing the quality of food. The role of the measuring apparatus is performed by the senses of smell, taste, and sight. Employing this technique makes it possible to specify hedonic qualities of foods, and the flavour and taste can be determined by assigning appropriate descriptors. In this research, samples of canola oil, olive oil, and mixtures of the two were analysed. In addition, samples were characterized by different thermal degradation levels. The parameter which was used for describing the quality of oils was palatability, determined on a scale from 1 to 5, with 5 representing the vegetable oil of highest quality. Based on the obtained results it can be observed that with an increase of the samples’ incubation temperature, the flavour of oils deteriorated. The tastiest oils were the ones incubated at *T*_inc_ = 20 °C. This applies to rapeseed oil, olive oil, and oil blend. These results confirm that the chemical compounds formed during oxidation, polymerization, and cyclization of oil could have a detrimental effect on the palatability. Furthermore, the analysis of flavour and taste of oils proves that with the increase of the temperature of incubation, undesirable characteristic of edible oils appear, such as rancid odour and taste. Change of oils’ taste and flavour during the heat treatment may be caused by the appearance of chemical compounds such as carboxylic acids (acidic taste), aldehydes (bitter, rancid, earthy), ketones (bitter, rancid), and short-chain hydrocarbons (bitter).

The samples were also analysed using the Rancimat test, which is used to evaluate the oxidation stability of edible oils. The measured value in this test is the time after which, during the heat treatment, thermal degradation products appear. These compounds are absorbed in deionized water, and the change of its electrical conductivity is measured in order to determine the induction time (*t*_induct_). Based on the obtained results, it can be observed that with the increase of the degree of thermal degradation, the thermal stability of oils decreases.

The most stable vegetable oil is olive oil, for which the induction times were the highest, both at 20, 60, and 100 °C. It was shown, that the addition of olive oil increases the thermal stability of canola oil. Blended oil samples incubated at 60 °C exhibit higher thermal stability than samples of canola oil incubated at the same temperature. It has also been shown that the oils incubated at temperatures above 140 °C have a short oxidation time, and are therefore not suitable as a frying medium. The results obtained during the sensory and Rancimat tests for canola oil, olive oil, and the oil blend are shown in Table 1.

**Table 1:** The results of Rancimat test and sensory analysis for canola, olive and blended oil incubated at five different temperatures for 24 h

Parameter	Incubation temperature (*T*_*inc*_)/°C				
	20	60	100	140	180
Canola oil					
*t*_induct_^a^/h	4.80 ± 0.38	0.080 ± 0.019	0.0333 ± 0.0077	0.0367 ± 0.017	0.0267 ± 0.0038
Palatability	3.50 ± 0.13	3.222 ± 0.059	1.833 ± 0.096	1.600 ± 0.093	1.222 ± 0.059
Flavour description	None, nutty, grassy	None, nutty, grassy	Grassy, rancid	Grassy, rancid	Grassy, rancid, acidic
Smell description	None, grassy	None, grassy	Grassy, rancid	Grassy, rancid	Grassy, rancid, earthy
Olive oil					
*t*_induct_/h	7.64 ± 0.82	7.08 ± 0.93	3.33 ± 0.28	0.0267 ± 0.0038	0.0300 ± 0.0067
Palatability	3.40 ± 0.11	3.78 ± 0.13	2.00 ± 0.11	1.636 ± 0.090	1.454 ± 0.070
Flavour description	None, grassy	Bittter, grassy, nutty	Bittter, grassy, nutty	Bittter, grassy, rancid	Bittter, grassy, rancid
Smell description	None, grassy	None, grassy	Grassy, acidic, earthy	Grassy, rancid	Grassy, rancid, earthy
Blended oil					
*t*_induct_/h	4.717 ± 0.156	4.533 ± 0.295	0.0350 ± 0.0047	0.0267 ± 0,0038	0.0267 ± 0.0038
Palatability	3.333 ± 0.104	3.538 ± 0.103	2.417 ± 0.089	1.538 ± 0.104	1.333 ± 0.066
Flavour description	None, grassy	Grassy, nutty	Grassy, nutty	Grassy, nutty, rancid	Rancid, nutty, bitter
Smell description	None, grassy	None, grassy, acidic	Grassy, acidic	Grassy, rancid, earthy	Rancid, bitter, earthy

^a^Induction time (Rancimat test result)

The proposed method of oil quality assessment is based on analysis of the edible oil’s headspace. For that purpose, ultra-fast GC-FID was used. Chromatograms obtained during the analysis were compared using fingerprint method and processed using chemometrics, in particular principal component analysis (PCA).

**Fig. 1:**
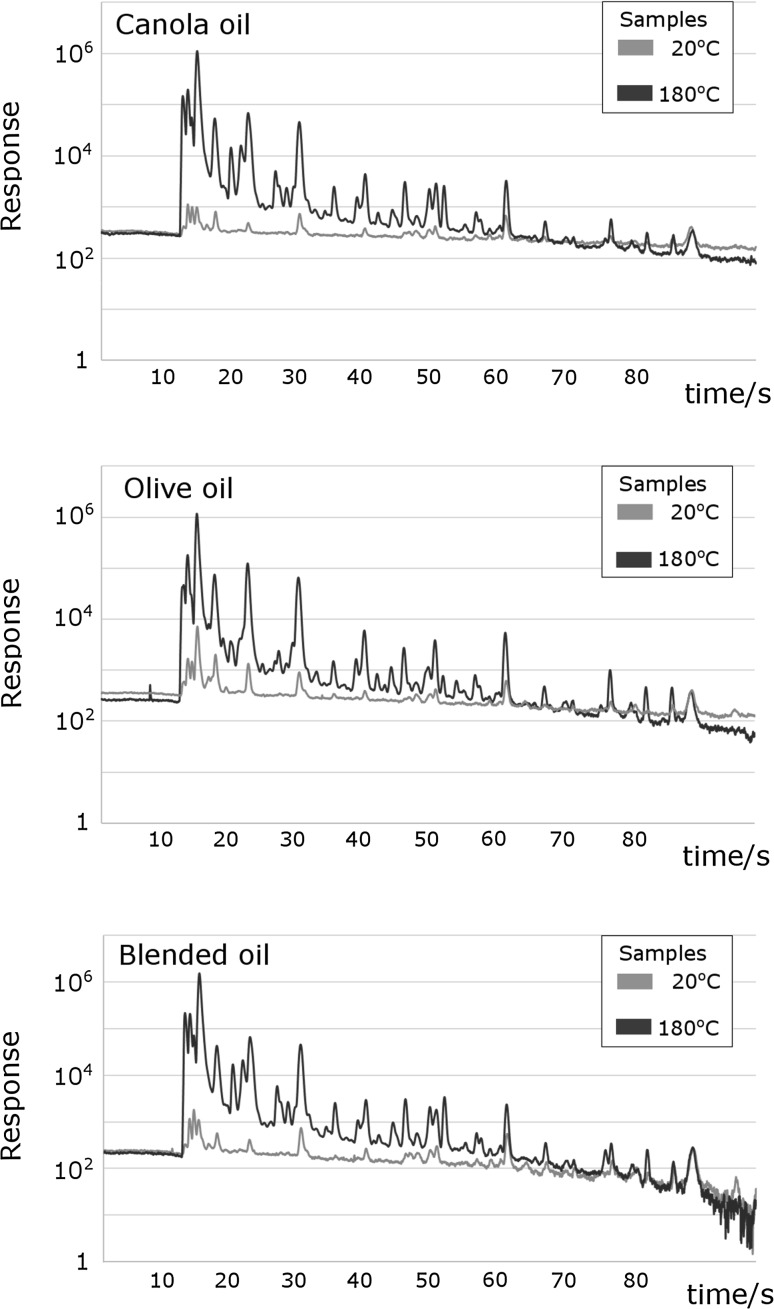
Chromatographic fingerprints of canola oil, olive oil, and blended oil samples incubated for 24 h at 20 and 180 °C

When the composition of the volatile fraction of oil samples is compared based on their unique fingerprint it can be observed that the thermal degradation process gives rise to numerous chemical compounds that were not present in non-degraded samples. Shown in Fig. 1 is a comparison of chromatograms obtained during the analysis of the volatile fraction of edible oils. By juxtaposing the chromatograms of oils incubated at 20 °C with the chromatograms of samples incubated at 180 °C it can be observed that a significant change occurs in the intensity of registered signals as well as in their multiplicity. It can be concluded, that the oil’s headspace composition changes both qualitatively and quantitatively.

**Fig. 2:**
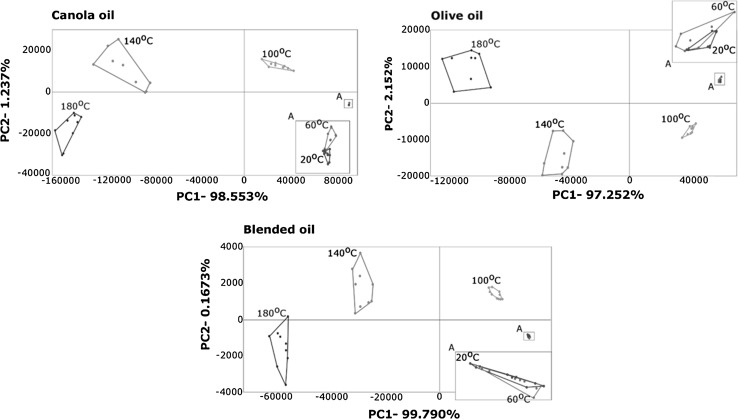
Classification of canola, olive, and blended oil incubating at five different temperatures at 24 h using PCA

Principal components analysis (PCA) may be used to classify samples differing in composition. Input values, which were used in the calculation, were the normalized peak areas. Vegetable oil samples incubated for 24 h at 20 °C were treated as reference samples. Plots in which the classification of oils of varying quality obtained using PCA is depicted are shown in Fig. 2. Based on these results it can be concluded that the thermal degradation of canola, olive, and blended oil samples incubated for 24 h at 60 °C was relatively low. The PCA score confirms Rancimat test results. In addition, it is believed that PCA can be successfully used for determining the thermal degradation progress of frying oil. The advantage of presented method, which can be used for assessing the quality of edible oils, is a relatively short time of a single analysis. It can be carried out in time less than in 2 min.

## Conclusion

Thermal treatment of vegetable oils results in decrease of their quality. Low-quality oils with unacceptable flavour and thermal stability, as well as undesirable composition cannot be used for frying or consumption. Currently used oil quality assessment methods have several limitations, due to which they cannot be used for routine tests. It is therefore necessary to develop a method for determining the quality of vegetable oils which is quick and easy to use. The advantage of the proposed method is the use of gas chromatography, which is a highly sensitive analytical technique that provides reproducible results.

Based on the presented research it can be stated that it is possible to determine the thermal degradation degree of canola and olive oil blend using the ultra-fast GC technique coupled with chemometrics. The obtained results were compared with reference methods, both sensory analysis and oxidation stability test using the Rancimat device. Sensory analysis was used to determine the hedonic quality of oil and identify main flavour and aroma descriptors of blended oil samples of varying quality. It was also demonstrated that the admixture of olive oil improves the usage parameters of canola oil. The oils blend displays higher oxidative stability and a lower susceptibility to thermal degradation than canola oil. The comparison of canola, olive, and blended oil samples’ fingerprints allows for the determination of thermal degradation of these oils. The proposed chemometric data analysis tool, principal component analysis, may be used to compare the composition of the headspace of oils in order to determine their quality. The employed method of evaluation of edible oil’s quality using ultra-fast gas chromatography is characterized by a relatively short time of a single analysis and no sample preparation step.

It is the author’s opinion that in the near future it will be possible to use the described method for evaluation of the degree of thermal degradation of vegetable oils as complementary to existing methods of assessing the quality of edible oils. In addition, it is important to continue research towards the development of a universal method for vegetable oils’ quality assessment. A method based on portable chemical sensors matrix coupled with chemometric tools, the so-called electronic nose, is planned [19].

## Experimental

### Sample preparation

Samples of refined canola oil, olive oil, and a commercially available oil blend (5% v/v olive oil), 5 cm^3^ each, were placed in 20 cm^3^ glass vials that were then sealed with a metal cap with a PTFE membrane. Samples were incubated for 24 h at five different temperatures: 20, 60, 100, 140, and 180 °C. As a result of this operation obtained oil samples were characterized by different thermal degradation levels and thus were of different quality. Depending on the incubation temperature, chemical reactions such as oxidation, cyclization, or polymerization have different intensities [17].

### Sensory analysis

Palatability was assessed in accordance with PN-A-86935:1996, which is Polish standard. In it, oil samples are evaluated on a five-grade scale. The highest score is assigned to oils with a pure, neutral flavour. On this scale, the palatability is defined as: 5—very good, 4—good, 3—satisfactory, 2—unsatisfactory, 1—bad. In addition, panellists were asked to write down the descriptors of flavour and aroma of oils. They were provided with a list of potential descriptors based on the work of Kiritsakis et al. [18]. The sensory panel consisted of 18 persons.

### Oxidative stability

In order to determine the oxidative stability of edible oils 893 Professional Biodiesel Rancimat (Metrohm AG, Herisau, Switzerland) was used. In accordance with ISO 6886:2006 during the measurement process samples were kept at 120 °C. The volumetric air flow was set to 20 dm^3^/h. The measured value in this method is the induction time, which is a value that can be related to the quality of oil. The longer the induction time, the better the quality of oil. Obtained data were processed using the StabNet software.

### Ultra-fast gas chromatography

The analysis of the volatile fraction of vegetable oils was conducted using ultra-fast chromatography device Heracles II equipped with HS100 autosampler (Alpha M.O.S., Toulouse, France). The gas chromatograph is equipped with two chromatography columns arranged in parallel. These chromatographic columns are: MXT-5, with crossbond diphenyl dimethyl polysiloxane stationary phase (10 m × 0.18 mm × 0.40 µm) and MXT-1701 with crossbond cyanopropylphenyl polysiloxane stationary phase (10 m × 0.18 mm × 0.40 µm) (Restek Corporation, Bellefonte, US). Hydrogen was used as a carrier gas. Detectors utilized in this device were two flame ionization detectors (μFIDs). Detailed information regarding the ultra-fast GC parameters is listed in Table 2.

**Table 2:** Ultra-fast GC-FID parameters used in the research

Autosampler					
Incubation time/s	Incubation temperature/°C	Syringe temperature/°C		Flushing time/s	Agitation speed/rpm
1200	40	100		90	500

Injector					
Injection volume/mm^3^	Injection speed/mm^3^/s		Injector temperature/°C		Vent/cm^3^/min
2500	250		200		30

Sorbent trap					
Trapping temperature/°C		Split/cm^3^/min		Trapping duration/s	
40		1		20	

Oven and detector					
Initial oven temperature/°C	Initial time/s	Rate/°C/s	Terminal temperature/°C	Terminal time/s	FID detectors temperature/°C
70	2	2.0	270	18	270

### Data processing

Principal component analysis was used to process the results of chromatographic analysis. PCA is an effective multivariate statistic method used in data classification [20]. The information provided in the input data is transformed into independent variables, or principal components. The first principal component covers the greatest number of input variables. The second principal component is orthogonal to the first and covers the largest possible number of the remaining variables. Plot of the principal components may be utilized to represent similarities and differences within the data set. Data analysis was performed using AlphaSoft v. 12.4 software.

## References

[1] Anwar F, Hussain AI, Iqbal S, Bhanger MI (2007). Food Chem.

[2] Choe E, Min DB (2007). J Food Sci.

[3] Farhoosh R, Kenari RE, Poorazrang H (2009). J Am Oil Chem Soc.

[4] De La Mata-Espinosa P, Bosque-Sendra JM, Bro R, Cuadros-Rodríguez L (2011). Talanta.

[5] Mamat H, Nor Aini I, Said M, Jamaludin R (2005). Food Chem.

[6] Cuesta C, Sánchez-Muniz FJ, Garrido-Polonio C, López-Varela S, Arroyo R (1993). J Am Oil Chem Soc.

[7] Zhang Q, Saleh ASM, Chen J, Shen Q (2012). Chem Phys Lipids.

[8] Martínez-Yusta A, Goicoechea E, Guillén MD (2014) Aldehydes after Prolonged Heating at Frying Temperature. Elsevier Inc

[9] Zarkovic N (2003). Mol Aspects Med.

[10] Willett WC, Stampfer MJ, Manson JE, Colditz GA, Speizer FE, Rosner BA, Sampson LA, Hannekens CH (1993). Lancet.

[11] Kummerow FA (2009). Atherosclerosis.

[12] Mozaffarian D (2006). Atheroscler Suppl.

[13] Polski Komitet Normalizacyjny (1996) PN-A-86935:1996, Oleje i tłuszcze roślinne oraz zwierzęce—Ocena sensoryczna smakowitości metodą punktową rafinowanych olejów i tłuszczów

[14] Polski Komitet Normalizacyjny (1993) PN-A-86926:1993, Tłuszcze roślinne jadalne—Oznaczanie liczby anizydynowej oraz obliczanie wskaźnika oksydacji tłuszczu Totox

[15] International Organization for Standardization (2006) ISO 6886:2006, Animal and vegetable fats and oils: determination of oxidative stability (accelerated oxidation test)

[16] European Union (1991) Commission Regulation (Eec) N^o^ 2568/91. Off J Eur Communities 1970

[17] Blumenthal MM (1991). Food Technol.

[18] Kiritsakis AK (1998). J Am Oil Chem Soc.

[19] Wardencki W, Biernacka P, Chmiel T, Dymerski T (2009). Proc ECOpole.

[20] Dymerski T, Gębicki J, Wardencki W, Namieśnik J (2014). Sensors.

